# *N*-Methyl-4-nitro­anilinium chloride

**DOI:** 10.1107/S1600536812024117

**Published:** 2012-05-31

**Authors:** Jian-Long Wang, Fu-Rong Zhou, Fei-Yun Wei

**Affiliations:** a School of Chemical Engineering and Environment, North University of China, Taiyuan, People’s Republic of China

## Abstract

The asymmetric unit of the title salt, C_7_H_9_N_2_O_2_^+^·Cl^−^, contains two independent cations and anions. In the crystal, each *N*-methyl-4-nitro­anilinium cation is linked to two Cl^−^ anions *via* N—H⋯Cl hydrogen bonds. π–π stacking is observed between the benzene rings of adjacent cations [centroid-to-centroid distances = 3.7684 (14) and 3.7917 (7) Å].

## Related literature

For applications of *N*-methyl-4-nitro­benzenamine, see: Bellamy & Sammour (1993 ▶); Sammour (1994 ▶); Williams & Friedlander (2000 ▶); Davies & Provatas (2006 ▶).
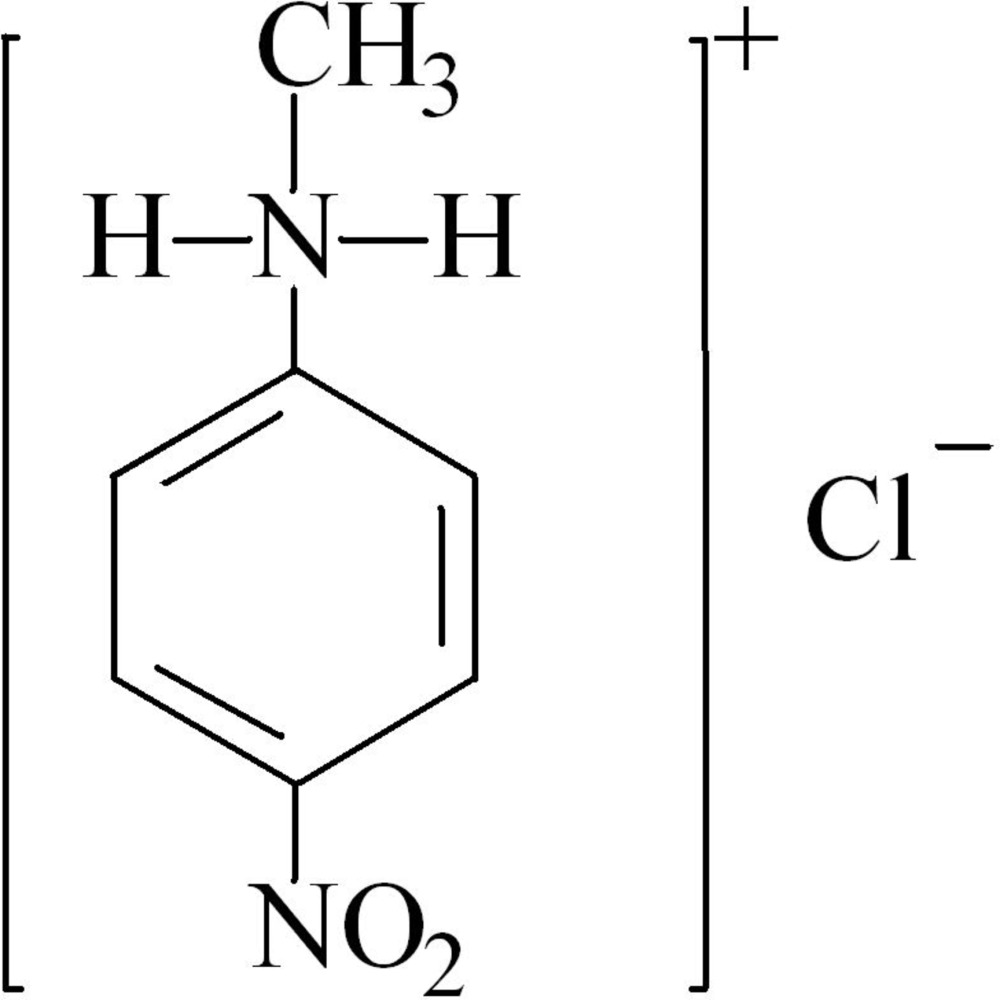


## Experimental

### 

#### Crystal data

C_7_H_9_N_2_O_2_^+^·Cl^−^
*M* *_r_* = 188.61
Monoclinic, 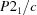

*a* = 7.0509 (14) Å
*b* = 19.120 (4) Å
*c* = 13.443 (3) Å
β = 95.20 (3)°
*V* = 1804.8 (6) Å^3^
*Z* = 8
Mo *K*α radiation
μ = 0.39 mm^−1^
*T* = 293 K
0.20 × 0.20 × 0.12 mm

#### Data collection

Rigaku Saturn diffractometer
Absorption correction: multi-scan (*ABSCOR*; Higashi, 1995 ▶) *T*_min_ = 0.927, *T*_max_ = 0.955
17869 measured reflections
4282 independent reflections
3022 reflections with *I* > 2σ(*I*)
*R*_int_ = 0.051

#### Refinement

*R*[*F* ^2^ > 2σ(*F* ^2^)] = 0.046
*wR*(*F* ^2^) = 0.124
*S* = 1.03
4282 reflections
236 parameters
4 restraints
H atoms treated by a mixture of independent and constrained refinement
Δρ_max_ = 0.24 e Å^−3^
Δρ_min_ = −0.27 e Å^−3^

### 

Data collection: *RAPID-AUTO* (Rigaku, 2000 ▶); cell refinement: *RAPID-AUTO*; data reduction: *CrystalStructure* (Rigaku/MSC, 2000 ▶); program(s) used to solve structure: *SHELXTL* (Sheldrick, 2008 ▶); program(s) used to refine structure: *SHELXTL*; molecular graphics: *SHELXTL*; software used to prepare material for publication: *SHELXTL*.

## Supplementary Material

Crystal structure: contains datablock(s) I, global. DOI: 10.1107/S1600536812024117/xu5532sup1.cif

Structure factors: contains datablock(s) I. DOI: 10.1107/S1600536812024117/xu5532Isup2.hkl

Supplementary material file. DOI: 10.1107/S1600536812024117/xu5532Isup3.cml

Additional supplementary materials:  crystallographic information; 3D view; checkCIF report

## Figures and Tables

**Table 1:** Hydrogen-bond geometry (Å, °)

*D*—H⋯*A*	*D*—H	H⋯*A*	*D*⋯*A*	*D*—H⋯*A*
N2—H2*A*⋯Cl2^i^	0.90 (1)	2.18 (1)	3.0702 (17)	170 (2)
N2—H2*B*⋯Cl1^i^	0.89 (1)	2.16 (1)	3.0482 (19)	173 (2)
N4—H4*A*⋯Cl2^ii^	0.90 (1)	2.15 (1)	3.0361 (18)	168 (2)
N4—H4*B*⋯Cl1	0.89 (1)	2.26 (1)	3.1157 (18)	163 (2)

Symmetry codes: (i) 
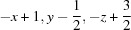
; (ii) 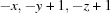.

**Fig. 1.**
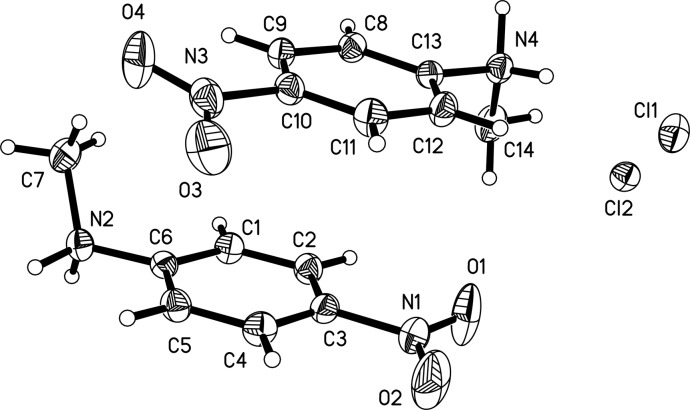
The molecular structure of the title compound.

**Fig. 2.**
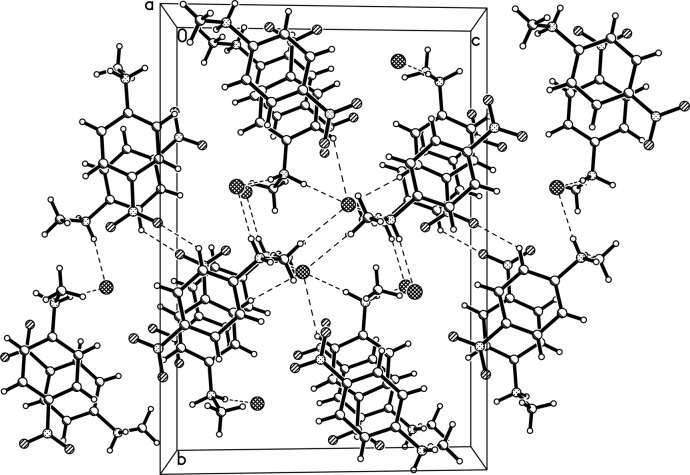
The packing of the title compound.

## supplementary crystallographic information

### Comment

As a stablizer, *N*-methyl-4-nitrobenzenamine is used in order to lengthen the useful service life of double-base and minimum smoke propellants (Bellamy & Sammour, 1993; Sammour, 1994; Williams & Friedlander, 2000). As a important ingredient, *N*-methyl-4-nitrobenzenamine can improve melt-cast explosive systems mechanical properties (Davies & Provatas, 2006). In order to research reaction properties, here we report the synthesis and the crystal structure of the title compound (Fig. 1).

The title compound consists of a *N*-(4-nitrophenyl)-methylammonium cation and a chloride anion. The hydrochloric acid deprotonated and the *N*-methyl-4-nitrobenzenamine accepts the proton to produce the protonated organic cation, namely *N*-(4-nitrophenyl)-methylammonium chloride. In the crystal structure, contains two cations and two anions. They are linked by N—H···Cl hydrogen bonds to form a three-dimensional complex network.

### Experimental

The title compound was synthesized by *N*-methyl-4-nitrobenzenamine and concentrated hydrochloric acid in acetone at room temperature. Single crystals suitable for X-ray diffraction were obtained by evaporation of a solution of the title compound in acetone at room temperature.

### Refinement

H atoms bonded to N atoms were located in a difference Fourier map and refined isotropically with bond restraint of N—H = 0.89 (2) Å. Other H atoms were positioned geometrically and treated as riding with C—H = 0.93–0.96 Å, and refined as riding with *U*_iso_(H) = 1.2–1.5*U*_eq_(C).

### Crystal data

**Table:**

C_7_H_9_N_2_O_2_^+^·Cl^−^	*F*(000) = 784
*M**_r_* = 188.61	*D*_x_ = 1.388 Mg m^−^^3^
Monoclinic, *P*2_1_/*c*	Mo *K*α radiation, λ = 0.71073 Å
Hall symbol: -P 2ybc	Cell parameters from 4042 reflections
*a* = 7.0509 (14) Å	θ = 2.6–27.9°
*b* = 19.120 (4) Å	µ = 0.39 mm^−^^1^
*c* = 13.443 (3) Å	*T* = 293 K
β = 95.20 (3)°	Block, yellow
*V* = 1804.8 (6) Å^3^	0.20 × 0.20 × 0.12 mm
*Z* = 8	

### Data collection

**Table:**

Rigaku Saturn diffractometer	4282 independent reflections
Radiation source: rotating anode	3022 reflections with *I* > 2σ(*I*)
Multilayer monochromator	*R*_int_ = 0.051
Detector resolution: 7.31 pixels mm^-1^	θ_max_ = 27.9°, θ_min_ = 2.6°
ω scans	*h* = −9→9
Absorption correction: multi-scan (*ABSCOR*; Higashi, 1995)	*k* = −25→25
*T*_min_ = 0.927, *T*_max_ = 0.955	*l* = −17→12
17869 measured reflections	

### Refinement

**Table:**

Refinement on *F*^2^	Secondary atom site location: difference Fourier map
Least-squares matrix: full	Hydrogen site location: inferred from neighbouring sites
*R*[*F*^2^ > 2σ(*F*^2^)] = 0.046	H atoms treated by a mixture of independent and constrained refinement
*wR*(*F*^2^) = 0.124	*w* = 1/[σ^2^(*F*_o_^2^) + (0.0651*P*)^2^] where *P* = (*F*_o_^2^ + 2*F*_c_^2^)/3
*S* = 1.03	(Δ/σ)_max_ = 0.001
4282 reflections	Δρ_max_ = 0.24 e Å^−^^3^
236 parameters	Δρ_min_ = −0.27 e Å^−^^3^
4 restraints	Extinction correction: *SHELXTL* (Sheldrick, 2008), Fc^*^=kFc[1+0.001xFc^2^λ^3^/sin(2θ)]^-1/4^
Primary atom site location: structure-invariant direct methods	Extinction coefficient: 0.073 (4)

### Special details

**Table:**

Geometry. All e.s.d.'s (except the e.s.d. in the dihedral angle between two l.s. planes) are estimated using the full covariance matrix. The cell e.s.d.'s are taken into account individually in the estimation of e.s.d.'s in distances, angles and torsion angles; correlations between e.s.d.'s in cell parameters are only used when they are defined by crystal symmetry. An approximate (isotropic) treatment of cell e.s.d.'s is used for estimating e.s.d.'s involving l.s. planes.
Refinement. Refinement of *F*^2^ against ALL reflections. The weighted *R*-factor *wR* and goodness of fit *S* are based on *F*^2^, conventional *R*-factors *R* are based on *F*, with *F* set to zero for negative *F*^2^. The threshold expression of *F*^2^ > σ(*F*^2^) is used only for calculating *R*-factors(gt) *etc*. and is not relevant to the choice of reflections for refinement. *R*-factors based on *F*^2^ are statistically about twice as large as those based on *F*, and *R*-factors based on ALL data will be even larger.

### Fractional atomic coordinates and isotropic or equivalent isotropic displacement parameters (Å^2^)

**Table:**

	*x*	*y*	*z*	*U*_iso_*/*U*_eq_	
O1	0.5920 (3)	0.47358 (8)	0.82213 (13)	0.0978 (7)	
O2	0.6814 (3)	0.46456 (9)	0.97433 (15)	0.1113 (9)	
O3	0.2318 (3)	0.28345 (10)	1.09445 (12)	0.0814 (6)	
O4	0.1062 (3)	0.20296 (9)	0.99778 (13)	0.0869 (6)	
N1	0.6303 (3)	0.43952 (9)	0.89526 (14)	0.0532 (5)	
N2	0.5910 (2)	0.14490 (8)	0.86838 (12)	0.0408 (4)	
N3	0.1557 (2)	0.26321 (10)	1.01448 (14)	0.0542 (5)	
N4	0.0461 (2)	0.45901 (8)	0.70054 (11)	0.0400 (4)	
C1	0.5619 (3)	0.26091 (10)	0.79113 (13)	0.0405 (4)	
H1	0.5343	0.2393	0.7295	0.049*	
C2	0.5707 (3)	0.33269 (10)	0.79740 (13)	0.0414 (4)	
H2	0.5485	0.3603	0.7406	0.050*	
C3	0.6131 (2)	0.36260 (9)	0.88971 (14)	0.0394 (4)	
C4	0.6468 (3)	0.32360 (10)	0.97592 (14)	0.0442 (5)	
H4	0.6757	0.3453	1.0374	0.053*	
C5	0.6365 (3)	0.25178 (10)	0.96866 (13)	0.0416 (4)	
H5	0.6580	0.2242	1.0256	0.050*	
C6	0.5942 (2)	0.22099 (9)	0.87655 (12)	0.0351 (4)	
C7	0.4023 (3)	0.11454 (11)	0.83074 (18)	0.0602 (6)	
H7A	0.3053	0.1324	0.8696	0.090*	
H7B	0.4074	0.0645	0.8365	0.090*	
H7C	0.3732	0.1272	0.7620	0.090*	
C8	0.0468 (3)	0.33800 (9)	0.76075 (13)	0.0388 (4)	
H8	0.0125	0.3233	0.6956	0.047*	
C9	0.0732 (3)	0.28977 (9)	0.83732 (14)	0.0411 (4)	
H9	0.0568	0.2422	0.8251	0.049*	
C10	0.1243 (3)	0.31429 (10)	0.93243 (13)	0.0402 (4)	
C11	0.1482 (3)	0.38398 (10)	0.95434 (14)	0.0474 (5)	
H11	0.1817	0.3987	1.0195	0.057*	
C12	0.1211 (3)	0.43201 (10)	0.87722 (13)	0.0423 (4)	
H12	0.1360	0.4796	0.8898	0.051*	
C13	0.0720 (2)	0.40829 (9)	0.78202 (12)	0.0347 (4)	
C14	0.1958 (3)	0.45515 (11)	0.62862 (15)	0.0557 (6)	
H14A	0.3184	0.4640	0.6634	0.084*	
H14B	0.1701	0.4896	0.5772	0.084*	
H14C	0.1948	0.4094	0.5991	0.084*	
Cl1	0.10531 (8)	0.61298 (3)	0.77271 (4)	0.05256 (19)	
Cl2	0.31979 (7)	0.57619 (3)	0.42629 (3)	0.04808 (18)	
H2A	0.632 (3)	0.1249 (11)	0.9271 (10)	0.062 (7)*	
H2B	0.684 (2)	0.1328 (12)	0.8311 (15)	0.075 (8)*	
H4A	−0.0708 (18)	0.4519 (12)	0.6697 (15)	0.068 (7)*	
H4B	0.041 (3)	0.5011 (7)	0.7277 (15)	0.064 (7)*	

### Atomic displacement parameters (Å^2^)

**Table:**

	*U*^11^	*U*^22^	*U*^33^	*U*^12^	*U*^13^	*U*^23^
O1	0.187 (2)	0.0365 (10)	0.0681 (12)	−0.0036 (11)	0.0040 (12)	0.0119 (8)
O2	0.181 (2)	0.0497 (11)	0.0926 (13)	−0.0070 (12)	−0.0467 (15)	−0.0217 (10)
O3	0.1046 (14)	0.0890 (14)	0.0489 (10)	0.0101 (11)	−0.0028 (9)	0.0215 (9)
O4	0.1261 (17)	0.0515 (11)	0.0827 (12)	0.0016 (10)	0.0070 (11)	0.0284 (9)
N1	0.0590 (11)	0.0368 (10)	0.0633 (12)	−0.0019 (8)	0.0029 (9)	−0.0075 (9)
N2	0.0475 (10)	0.0353 (9)	0.0395 (9)	0.0044 (7)	0.0038 (7)	0.0004 (7)
N3	0.0558 (11)	0.0538 (12)	0.0544 (11)	0.0097 (9)	0.0121 (9)	0.0178 (9)
N4	0.0442 (9)	0.0350 (9)	0.0396 (9)	−0.0033 (7)	−0.0021 (7)	0.0040 (7)
C1	0.0491 (11)	0.0400 (11)	0.0317 (9)	−0.0016 (8)	0.0002 (8)	−0.0022 (7)
C2	0.0455 (11)	0.0402 (11)	0.0380 (10)	0.0005 (8)	0.0019 (8)	0.0049 (8)
C3	0.0381 (10)	0.0339 (10)	0.0463 (11)	−0.0009 (7)	0.0047 (8)	−0.0031 (8)
C4	0.0525 (12)	0.0453 (11)	0.0341 (10)	−0.0021 (9)	−0.0003 (8)	−0.0069 (8)
C5	0.0477 (11)	0.0428 (11)	0.0337 (9)	0.0026 (8)	0.0001 (8)	0.0035 (8)
C6	0.0331 (9)	0.0339 (9)	0.0381 (10)	0.0019 (7)	0.0026 (7)	−0.0008 (7)
C7	0.0611 (14)	0.0381 (12)	0.0789 (16)	−0.0080 (10)	−0.0066 (12)	−0.0052 (10)
C8	0.0414 (10)	0.0345 (10)	0.0400 (10)	−0.0010 (8)	0.0009 (8)	−0.0033 (8)
C9	0.0399 (10)	0.0312 (10)	0.0526 (12)	0.0011 (7)	0.0065 (8)	0.0024 (8)
C10	0.0398 (10)	0.0397 (11)	0.0416 (10)	0.0034 (8)	0.0067 (8)	0.0108 (8)
C11	0.0588 (13)	0.0462 (12)	0.0364 (10)	0.0007 (9)	−0.0010 (9)	0.0004 (8)
C12	0.0534 (12)	0.0314 (10)	0.0408 (10)	−0.0031 (8)	−0.0028 (8)	−0.0024 (8)
C13	0.0332 (9)	0.0340 (9)	0.0366 (9)	−0.0004 (7)	0.0015 (7)	0.0048 (7)
C14	0.0711 (15)	0.0500 (13)	0.0483 (12)	−0.0070 (10)	0.0177 (10)	0.0066 (10)
Cl1	0.0642 (4)	0.0381 (3)	0.0568 (3)	−0.0017 (2)	0.0129 (2)	0.0025 (2)
Cl2	0.0535 (3)	0.0514 (3)	0.0381 (3)	−0.0033 (2)	−0.0030 (2)	−0.0018 (2)

### Geometric parameters (Å, º)

**Table:**

O1—N1	1.190 (2)	C4—C5	1.378 (3)
O2—N1	1.191 (2)	C4—H4	0.9300
O3—N3	1.220 (2)	C5—C6	1.379 (2)
O4—N3	1.218 (2)	C5—H5	0.9300
N1—C3	1.477 (2)	C7—H7A	0.9600
N2—C6	1.459 (2)	C7—H7B	0.9600
N2—C7	1.497 (3)	C7—H7C	0.9600
N2—H2A	0.901 (9)	C8—C9	1.382 (2)
N2—H2B	0.891 (10)	C8—C13	1.382 (2)
N3—C10	1.475 (2)	C8—H8	0.9300
N4—C13	1.462 (2)	C9—C10	1.379 (3)
N4—C14	1.496 (3)	C9—H9	0.9300
N4—H4A	0.898 (10)	C10—C11	1.372 (3)
N4—H4B	0.885 (10)	C11—C12	1.385 (3)
C1—C2	1.376 (3)	C11—H11	0.9300
C1—C6	1.380 (2)	C12—C13	1.372 (2)
C1—H1	0.9300	C12—H12	0.9300
C2—C3	1.374 (2)	C14—H14A	0.9600
C2—H2	0.9300	C14—H14B	0.9600
C3—C4	1.380 (3)	C14—H14C	0.9600
			
O1—N1—O2	123.02 (19)	C5—C6—C1	121.13 (16)
O1—N1—C3	119.47 (17)	C5—C6—N2	119.60 (15)
O2—N1—C3	117.52 (18)	C1—C6—N2	119.23 (15)
C6—N2—C7	114.80 (14)	N2—C7—H7A	109.5
C6—N2—H2A	110.9 (14)	N2—C7—H7B	109.5
C7—N2—H2A	109.6 (14)	H7A—C7—H7B	109.5
C6—N2—H2B	107.1 (16)	N2—C7—H7C	109.5
C7—N2—H2B	112.7 (16)	H7A—C7—H7C	109.5
H2A—N2—H2B	101 (2)	H7B—C7—H7C	109.5
O4—N3—O3	123.90 (19)	C9—C8—C13	119.26 (16)
O4—N3—C10	117.86 (18)	C9—C8—H8	120.4
O3—N3—C10	118.23 (19)	C13—C8—H8	120.4
C13—N4—C14	113.82 (15)	C10—C9—C8	118.03 (17)
C13—N4—H4A	107.0 (14)	C10—C9—H9	121.0
C14—N4—H4A	111.5 (14)	C8—C9—H9	121.0
C13—N4—H4B	107.5 (14)	C11—C10—C9	123.10 (17)
C14—N4—H4B	111.6 (15)	C11—C10—N3	118.39 (17)
H4A—N4—H4B	104.9 (19)	C9—C10—N3	118.50 (17)
C2—C1—C6	119.82 (16)	C10—C11—C12	118.52 (17)
C2—C1—H1	120.1	C10—C11—H11	120.7
C6—C1—H1	120.1	C12—C11—H11	120.7
C3—C2—C1	118.37 (16)	C13—C12—C11	119.01 (17)
C3—C2—H2	120.8	C13—C12—H12	120.5
C1—C2—H2	120.8	C11—C12—H12	120.5
C2—C3—C4	122.68 (17)	C12—C13—C8	122.08 (16)
C2—C3—N1	118.04 (16)	C12—C13—N4	118.90 (16)
C4—C3—N1	119.23 (16)	C8—C13—N4	119.02 (15)
C5—C4—C3	118.36 (16)	N4—C14—H14A	109.5
C5—C4—H4	120.8	N4—C14—H14B	109.5
C3—C4—H4	120.8	H14A—C14—H14B	109.5
C4—C5—C6	119.64 (16)	N4—C14—H14C	109.5
C4—C5—H5	120.2	H14A—C14—H14C	109.5
C6—C5—H5	120.2	H14B—C14—H14C	109.5
			
C6—C1—C2—C3	0.4 (3)	C13—C8—C9—C10	−0.1 (3)
C1—C2—C3—C4	−0.1 (3)	C8—C9—C10—C11	0.7 (3)
C1—C2—C3—N1	177.47 (17)	C8—C9—C10—N3	−179.08 (16)
O1—N1—C3—C2	6.2 (3)	O4—N3—C10—C11	168.9 (2)
O2—N1—C3—C2	−174.3 (2)	O3—N3—C10—C11	−12.1 (3)
O1—N1—C3—C4	−176.2 (2)	O4—N3—C10—C9	−11.3 (3)
O2—N1—C3—C4	3.3 (3)	O3—N3—C10—C9	167.65 (18)
C2—C3—C4—C5	−0.3 (3)	C9—C10—C11—C12	−0.5 (3)
N1—C3—C4—C5	−177.77 (17)	N3—C10—C11—C12	179.22 (17)
C3—C4—C5—C6	0.3 (3)	C10—C11—C12—C13	−0.1 (3)
C4—C5—C6—C1	0.0 (3)	C11—C12—C13—C8	0.6 (3)
C4—C5—C6—N2	177.53 (17)	C11—C12—C13—N4	−179.17 (17)
C2—C1—C6—C5	−0.4 (3)	C9—C8—C13—C12	−0.5 (3)
C2—C1—C6—N2	−177.88 (17)	C9—C8—C13—N4	179.30 (16)
C7—N2—C6—C5	118.4 (2)	C14—N4—C13—C12	111.3 (2)
C7—N2—C6—C1	−64.1 (2)	C14—N4—C13—C8	−68.5 (2)

### Hydrogen-bond geometry (Å, º)

**Table:**

*D*—H···*A*	*D*—H	H···*A*	*D*···*A*	*D*—H···*A*
N2—H2*A*···Cl2^i^	0.90 (1)	2.18 (1)	3.0702 (17)	170 (2)
N2—H2*B*···Cl1^i^	0.89 (1)	2.16 (1)	3.0482 (19)	173 (2)
N4—H4*A*···Cl2^ii^	0.90 (1)	2.15 (1)	3.0361 (18)	168 (2)
N4—H4*B*···Cl1	0.89 (1)	2.26 (1)	3.1157 (18)	163 (2)

Symmetry codes: (i) −*x*+1, *y*−1/2, −*z*+3/2; (ii) −*x*, −*y*+1, −*z*+1.
